# Causes of mortality and morbidity in free-ranging mustelids in Switzerland: necropsy data from over 50 years of general health surveillance

**DOI:** 10.1186/s12917-018-1494-0

**Published:** 2018-06-19

**Authors:** E. Akdesir, F. C. Origgi, J. Wimmershoff, J. Frey, C. F. Frey, M.-P. Ryser-Degiorgis

**Affiliations:** 1 Centre for Fish and Wildlife Health (FIWI), Vetsuisse Faculty, University of Bern, Länggassstrasse 122, Postfach, 3001 Bern, Switzerland; 2 Institute of Veterinary Bacteriology, Vetsuisse Faculty, University of Bern, Länggassstrasse 122, Postfach, 3001 Bern, Switzerland; 3 Institute of Parasitology, Vetsuisse Faculty, University of Bern, Länggassstrasse 122, Postfach, 3001 Bern, Switzerland

**Keywords:** Amyloidosis, Bacteria, Badger, Canine distemper, Histoplasmosis, Marten, Parasites, Pathology, Virus, Sarcoptic mange

## Abstract

**Background:**

Although mustelids occur worldwide and include a wide range of species, little is known about the diseases affecting them. Mustelids have regularly been submitted for post mortem investigation in the framework of the program for general wildlife health surveillance in Switzerland, which has been in place for nearly 60 years. We performed a retrospective analysis of the necropsy reports on mustelids submitted to the diagnostic service of the University of Bern. The aims of this study were to present an overview of the causes of mortality and morbidity observed in these carnivores, to assess differences among species, to assess changes in disease detection over the study period, and to describe the pathology of selected diseases.

**Results:**

Five hundred and sixty-six reports from 1958 to 2015 were analyzed. Most animals were stone martens (*Martes foina,* 46%) and badgers (*Meles meles*, 44%); the remaining species were polecats (*Mustela putorius*, 4.7%), pine martens (*Martes martes*, 2%), stoats (*Mustela erminea*, 1.4%), weasels (*Mustela nivalis*, 0.8%) and otters (*Lutra lutra*, 0.3%). Infectious diseases (*n* = 262) were frequent and were mostly bacterial or viral; non-infectious conditions (*n* = 169) were less common and were mostly traumatic or due to metabolic disorders. The most frequent diagnoses included distemper (75% were badgers), amyloidosis (96% were martens), bacterial respiratory infections (all mustelids), biting lice (badgers only) and pulmonary and gastro-intestinal helminths (all species). Less frequent diseases included histoplasmosis (badgers only), aspergillosis, toxoplasmosis, hepatozoonosis, and sarcoptic mange. Lesions due to infection with distemper virus were primarily appreciated in the respiratory tract and central nervous system; they presented species-specific characteristics such as necrosis in the ependyma in badgers and absence of syncytia in stone martens. Amyloidosis in martens was multisystemic in most cases and included both AA and AL amyloidosis; the main macroscopic change was severe splenomegaly.

**Conclusion:**

Infectious diseases were the most frequent causes of morbidity and mortality of mustelids, with marked species-specific differences. Lung and skin were the most commonly affected organs. Contagious diseases such as canine distemper, sarcoptic mange and rabies in mustelids showed a similar temporal pattern as in red foxes (*Vulpes vulpes*), suggesting pathogen spillovers from foxes to mustelids.

## Background

Mustelids represent the largest family within the Carnivora order, consisting of 67 small to medium sized species with an elongated, slender body and short extremities (although there are some exceptions to this morphology). Mustelids occur worldwide, except in Australia and Antarctica [1, 2]. While some species are prolific and are considered pests because of the physical damage they cause to human property [3] or because of the pathogens they may carry [4, 5], others are experiencing population declines or are even listed as endangered [2].

The biology of a number of free-ranging mustelid species has been extensively studied but little is known about the causes of mortality in this family. Available data about diseases are generally limited to selected infections, such as bovine tuberculosis in the Eurasian badger (*Meles meles*) [4, 6, 7], rabies in multiple species [5], or canine distemper [8–11] which has caused a severe population decline in black footed ferrets (*Mustela nigripes*) [12]. Aleutian disease has been investigated mostly in farmed mink (*Neovison vison*) but it may also be relevant to free-ranging mustelids such as the European mink (*Mustela lutreola*) [13–18]. Ectoparasites and endoparasites have been studied in free-ranging populations [19–23] but only rarely as causes of significant pathological lesions [24]. Reports on histoplasmosis in badgers and amyloidosis in martens (*Martes* spp.) are restricted to single cases or small case series [25–30]. A more recent study on stoats (*Mustela erminea*), weasels (*Mustela nivalis*) and polecats (*Mustela putorius*) presented data collected over 15 years but it was limited to respiratory disorders [31]. Thus, overviews on causes of mortality and morbidity in free-ranging mustelids are still lacking.

It has now been recognized that knowledge arising from general wildlife health surveillance is an important component of the One Health concept (i.e., the concept that the health of humans, animals and ecosystems is interconnected and that a coordinated, collaborative, multidisciplinary and cross-sectoral approach is required to address risks originating at the animal-human-ecosystems interface) [32–34]. General wildlife health surveillance is also recognized as being integral to wildlife conservation [35]. Carcasses of free-ranging wild animals submitted for necropsy in the framework of general surveillance are usually not representative for the whole population due to numerous factors influencing carcass submission. However, this type of surveillance delivers essential information on disease occurrence because pathological investigations are not restricted to the detection of selected causative agents but, as far as possible, document all disease processes affecting the animals investigated [36, 37]. Unfortunately, such information is only rarely published or publicly disseminated. Health surveillance, by definition, should not only include data collection but also data analysis and communication [37].

Mustelids can be found everywhere in Switzerland, but abundance, distribution and protection status vary among species. The Eurasian badger and stone marten (*Martes foina*) are common and can be hunted. The pine marten (*Martes martes*), stoat, weasel and polecat are much less abundant and have been protected species since 1978. The European fish otter (*Lutra lutra*) vanished from Switzerland in 1950; it has been protected by law since 1952 and a few individuals are now slowly recolonizing water streams in the midlands and the south-eastern part of the country [38]. A few ferrets (*Mustela putorius furo*) and American minks (*Neovison vison*) escaped from captivity in the past but have apparently not established free-ranging populations [1, 39, 40].

Wildlife health surveillance in Switzerland dates back to the 1950s. Several institutions have been involved in wildlife disease investigations but for the past two decades the majority of cases have been submitted to the Centre for Fish and Wildlife Health (FIWI) at the University of Bern, which is the national diagnostic laboratory for wildlife diseases [41]. Free-ranging mustelids have regularly been submitted for post mortem investigation since 1958.

This study aimed to increase the knowledge on causes of death and diseases of free-ranging European mustelids. Specifically, we wanted 1) to provide an overview of the causes of mortality and morbidity observed in free-ranging mustelids in Switzerland over half a century of general health surveillance; 2) to assess differences among species and potential changes in disease detection over time; and 3) to describe the pathological picture of selected diseases.

## Methods

### Study area

Switzerland (41,285 km^2^) is composed of 26 political units (cantons) of various sizes and three main bioregions: the Jura Mountains, shaped by forests and pastures; the highly urbanized Swiss Midlands; and the Alps, of which a large part is located above the timberline [42]. The Principality of Liechtenstein (160 km^2^) is adjacent to the eastern Swiss border and affiliated with the Swiss veterinary services (status similar to that of a canton). Depending on the hunting system of the canton, either hunters or officially appointed game wardens are in charge of wildlife health surveillance in the field [41, 43].

### Study material

The study included all available necropsy reports of mustelids submitted to the wildlife group of the University of Bern (referred to as FIWI) from 1958 to 2015 (except the missing archive of 1960, 1969 and 1975-1977). There were 566 cases from seven species (badger, stone marten, pine marten, polecat, weasel, stoat and otter; Table 1) originating from 24 of the 26 Swiss cantons and from Liechtenstein.

**Table 1:** Free-ranging mustelids submitted to the FIWI for pathological examination, Switzerland, 1958-2015

Species	Total	Adult			Young			Unknown age		
		F	M	U	F	M	U	F	M	U
Badger	249	73	86	5	21	31	3	14	11	2
Stone marten	262	62	99	2	29	25	4	11	18	9
Pine marten	13	4	4	1	1	0	0	0	2	1
Polecat	27	4	8	0	5	4	3	1	2	0
Stoat	8	1	3	0	1	2	0	0	1	0
Weasel	5	0	0	0	1	0	0	1	2	1
Otter	2	1	1	0	0	0	0	0	0	0
Total	566	145	201	8	58	62	10	27	36	13

*F* Female, *M* Male, *U* Unknown

Young: juveniles and subadults

**Fig. 1:**
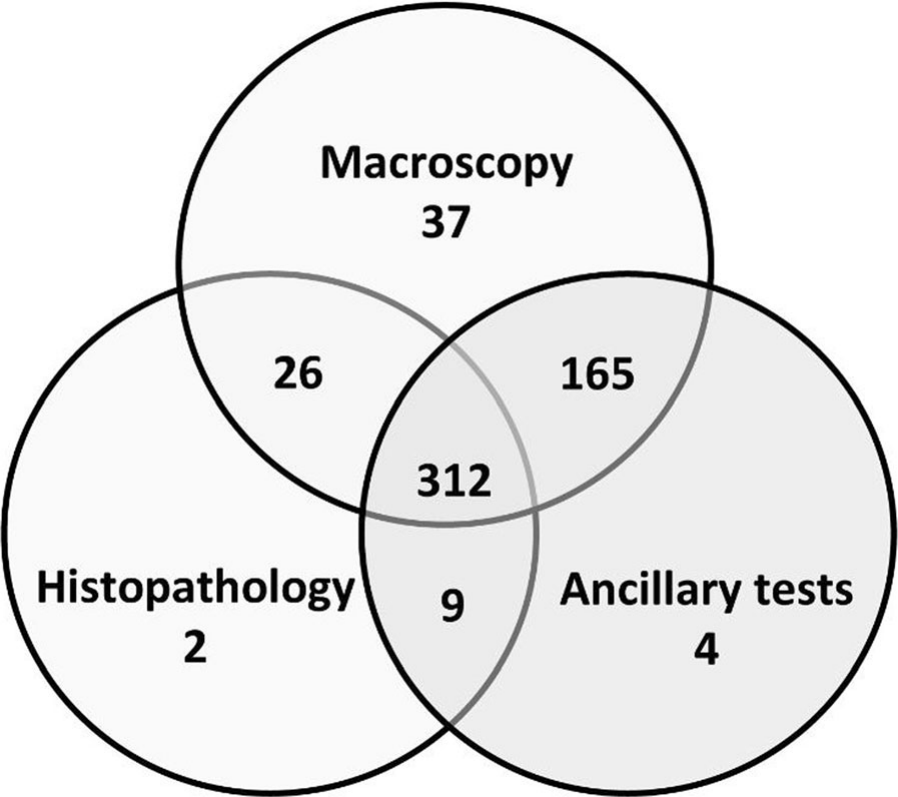
Investigations performed on the free-ranging mustelids submitted to the FIWI, Switzerland, 1958 to 2015. Ancillary tests: bacteriological, virological, parasitological and/or toxicological analyses

Macroscopic examination was carried out either on whole carcasses (*n* = 550), or on single organs selected by the submitters (*n* = 16); histopathology was performed in most cases (*n* = 353). Tissue processing and staining methods were performed according to the standard protocols of the Institute of Animal Pathology of the University of Bern [43]. Additional diagnostic testing was conducted as indicated by case history and gross necropsy findings. In 490 cases at least one of the following additional examinations was performed: parasitology, bacteriology, virology and/or toxicology (Fig. 1). Laboratory protocols as well as completeness and accuracy of the necropsy reports varied throughout the study period due to administrative structural changes, personnel experience and technique availability [43].

Parasitological examinations (*n* = 300) included coprological analyses (flotation, sedimentation and Baermann-funnel; *n* = 271), digestion assay for *Trichinella* spp. (*n* = 41) [44], macroparasite identification (proglottids, helminth larvae and adults) [45, 46], and until 1992 washouts of gastrointestinal contents with subsequent sieving to retrieve parasites. Sarcoptic mange was diagnosed based on presentation of typical skin lesions together with the identification of intralesional *Sarcoptes scabiei* in affected skin samples. Light microscopy was used to examine skin scraping material and/or live mites isolated from skin specimens (by placing the skin specimen in petri dishes under a light source [47]). Mites were identified morphologically [48].

Virological analyses (*n* = 340) were performed for canine distemper virus (CDV) and rabies virus. Rabies testing was conducted at the Swiss Rabies Centre (Institute of Veterinary Virology, University of Bern) by immunofluorescence assay (IFA). At the time of the rabies epidemic (1967 to 1997), cases suspicious for rabies were submitted directly to the Swiss Rabies Centre. Positive cases were not forwarded to the FIWI for analysis and were therefore not included in this study. Until 1992, CDV testing was also performed by IFA at the Institute of Veterinary Virology of the University of Bern. From 1993 onwards, immunohistochemistry (IHC, *n* = 42) [49] was used to test for CDV; from 2010 onwards this was increasingly replaced by polymerase chain reaction (PCR; *n* = 29) [10].

Bacteriological analyses (*n* = 300) were carried out using standard cultivation and identification by biochemical strips (API 20 E/NE) and more recently by Matrix-Assisted Laser Desorption Ionization – Time Of Flight (MALDI-TOF). Fungi were cultured on Sabouraud-Dextrose Agar [43]. *Histoplasma capsulatum* was presumptively diagnosed based on characteristic morphological features on histology [50]. Samples from four badgers from areas at risk for bovine tuberculosis were tested by PCR for the presence of mycobacteria of the *Mycobacterium tuberculosis* complex at the Swiss national reference laboratory for tuberculosis (Institute for Veterinary Bacteriology, University of Zurich) according to established protocols [51]. Of these four badgers, one animal from the canton of Grisons, 2010, presented with multiple purulent skin wounds; and three badgers from the canton of Fribourg were culled for targeted testing in 2013 and were apparently healthy.

Toxicological examinations were documented in four cases with suspected poisoning (two martens examined in 1958 and 1991; a stoat examined in 1989; and a badger examined in 1990). Unfortunately, documentation did not specify either the laboratory or the analytic methods.

### Data management

Animal data, case history (in particular the mention of behavioral changes such as aggression or fearlessness in the presence of people or domestic animals, apathy and diurnal activity), submitted material, submitter details, results of investigations and name of the investigators were digitalized in an Excel table (Microsoft Excel 2010, Microsoft Corporation, Redmond, Washington, USA). Martens without species specification (*Martes* sp., *n* = 15) were counted as stone martens because this is the most common marten species in Switzerland and often simply referred to as “marten”.

Animals were classified as either young (including animals mentioned as either juvenile or subadult on the reports) or adult. In absence of age information (*n* = 102), age was estimated (*n* = 31) by comparing available animal data (body weight, condition, sex, season, age) with those of other submitted cases of known age groups and with published reference values [1, 52]. When data were insufficient, the age group remained unknown (*n* = 71). Seasons referred to calendar months (winter: January-March; spring: April-June; summer: July-September; Fall: October-December).

Maps were drawn with the free software QGIS 2.8 Wien [53] using the submitted (*n* = 120) or estimated (*n* = 400) coordinates. Coordinates were estimated using the location information provided by the submitter and the website of the Federal Office for Swiss Topography [54]. Forty-six cases with insufficient location information were not mapped.

The “main diagnosis” was defined as the most likely cause of death (if the animal was found dead) or as the most likely cause of the clinical signs that had motivated the culling of the animal. Main diagnoses were classified based on etiology. Additional findings were recorded separately but categorized according to the same scheme as the main diagnoses [43, 55].

### Retrospective investigations on canine distemper

Histopathological changes consistent with canine distemper (CD; interstitial pneumonia or inflammation of the central nervous system [10]) were mentioned in the necropsy report of 25 out of 45 cases with reported abnormal behavior but without etiological diagnosis and without mention of CDV testing. Based on the availability of archival paraffin blocks and tissue preservation, 10 of these 25 cases were later selected to be tested for CDV by PCR. They included six cases from 1993 and one case each from 1997, 1998, 2000, and 2005. RNA-extraction from paraffin blocks, RT-PCR and gel electrophoresis steps were performed as previously described [10]. Additionally, sections of brain and lung tissues of cases confirmed as CDV positive either by PCR or IHC (*n* = 45, from 1999 to 2015) were evaluated based on previously published criteria [10] with some additions.

To assess differences in disease detection and pathological features, two time periods were distinguished, namely before and after the large distemper epidemic that hit Switzerland in 2009 and subsequently expanded over the country from east to west, affecting mainly red foxes [10].

### Retrospective investigations on amyloidosis

Tissue sections of spleen and kidneys from eight martens affected by amyloidosis for which archival material was available were stained with Congo red (with and without potassium permanganate pre-treatment) and examined for birefringence under polarized light to determine the type of amyloid deposits. Amyloidosis was classified as AA amyloidosis in absence of birefringence and as non-AA amyloidosis in presence of birefringence following potassium permanganate pre-treatment [30, 56].

### Statistical analyses

Differences among species, age groups, seasons and study periods in our investigation material were assessed using either the chi-square test or the two-tailed Fisher’s exact test. The tests were run with NCSS 2001 software (Hintze, J., 2001; NCSS, Kaysville, Utah, USA [57]). Level of significance was set at *P* < 0.05.

## Results

### Submitted material

The submitted species included mainly stone martens (46%) and badgers (44%); a few polecats (4.7%), pine martens (2%), stoats (1.4%) and weasels (0.8%); and two otters (0.3%; Table 1). Adults were more numerous than young animals in all species, except for the polecat in which both groups were equal. There were generally more males than females (53 and 41% of all animals, respectively).

**Fig. 2:**
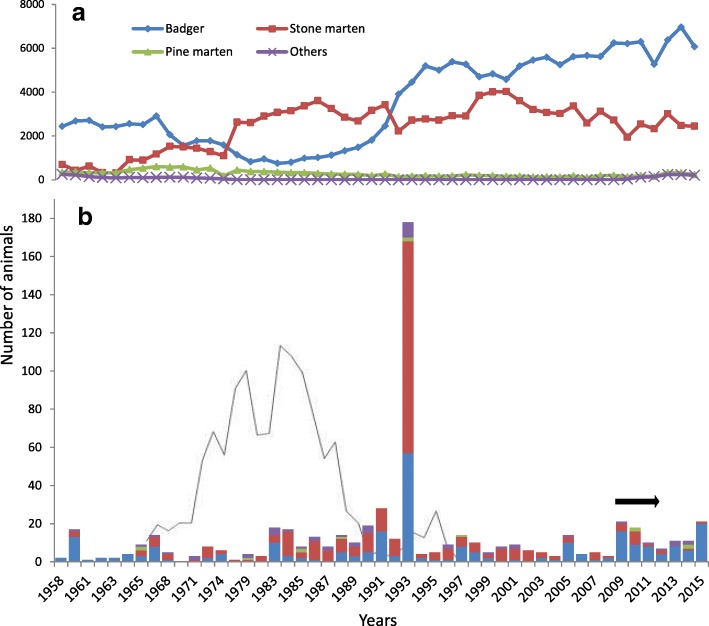
Free-ranging mustelids found dead, culled or hunted in Switzerland from 1958 to 2015. Diagram **a** shows the total number of dead mustelids, including those shot and found dead, which were recorded in Switzerland from 1958 to the winter 2014/15 (national hunting statistics). Diagram **b** shows the number of cases submitted to the FIWI from 1958 to 2015 (animals included in the present study), using the same color code for different mustelid species as in Diagram a. The black line in Diagram b represents the number of mustelids diagnosed positive for rabies at the Swiss Rabies Center. Rabies investigations began in 1967 and cases were recorded until 1997, when the disease was eradicated from Switzerland. The horizontal black arrow indicates the years of the national epidemic of canine distemper

**Fig. 3:**
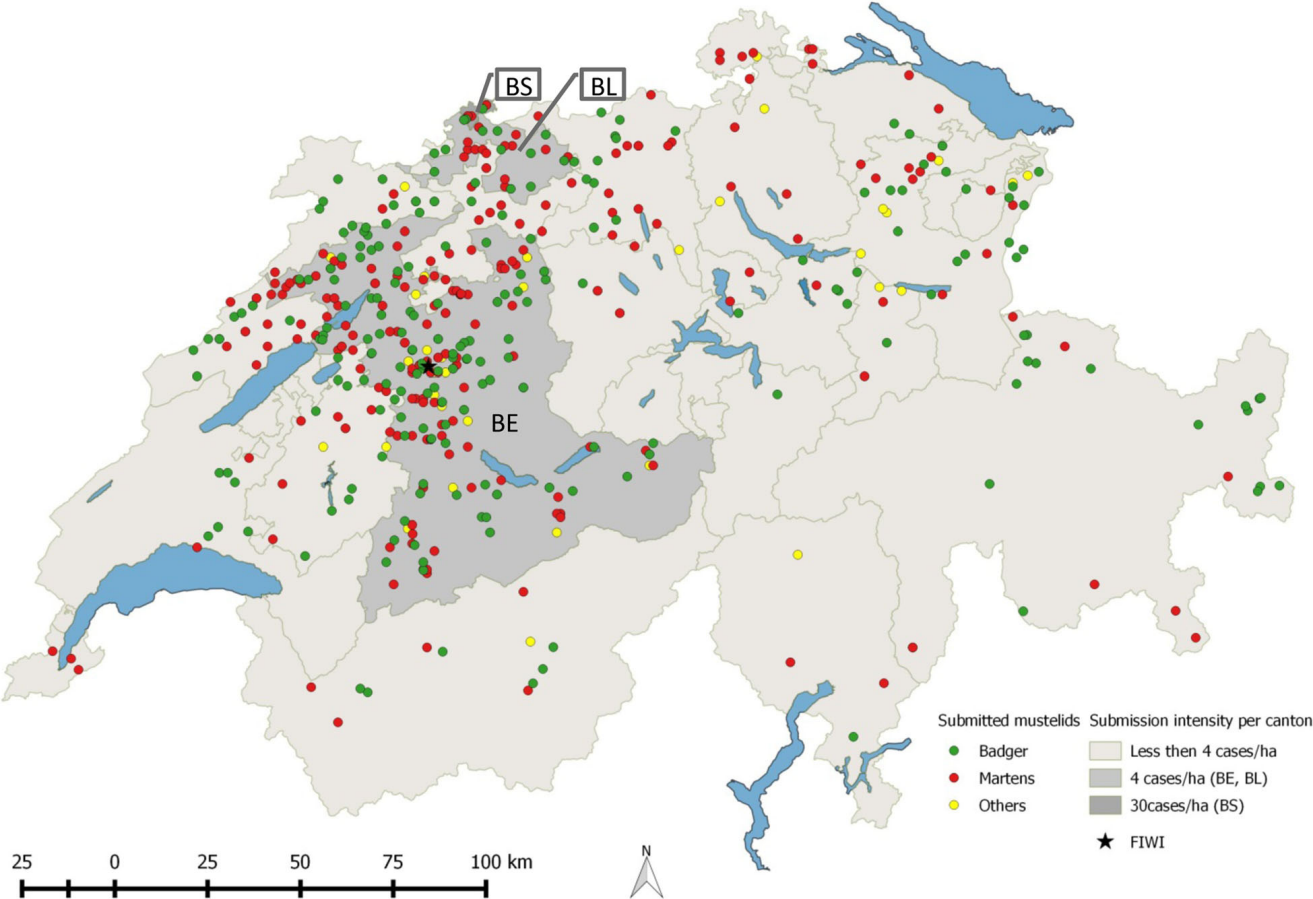
Map of Switzerland depicting the geographical origin of the mustelids submitted to the FIWI, 1958-2015. Different mustelid species are indicated by dots of different colors: Green: Badgers, Red: Martens (including stone marten *Martes foina,* and pine marten *Martes martes*), Yellow: Others (polecat *Mustela putorius*, stoat *Mustela ermine*, weasel *Mustela nivalis* and Eurasian otter *Lutra lutra*). Areas with different shades of grey illustrate the case density or submission intensity per Swiss canton (number of submitted cases per ha). BE: Canton Bern; BS: Canton Basel-Stadt; BL: Canton Basel-Landschaft

Case numbers were significantly higher in spring (*n* = 206) and summer (*n* = 209) than in autumn (*n* = 91; *P* < 0.001) and winter (*n* = 57; *P* < 0.001) in all species. Besides a marked peak of cases in 1993 (*n* = 188, 33%), yearly case numbers varied from 2 to 28 (Fig. 2) with a mean of about eight animals per year. Nearly half of the submissions originated from the canton of Bern (45%) but the number of cases per hectare was the highest in the canton of Basel-Stadt (30 cases/ha, 13 cases), followed by Basel-Landschaft (4 cases/ha, 24 cases) and Bern (4 cases/ha, 247 cases; Fig. 3). In terms of bioregions, the number of submissions was lowest from the Alps.

Submission was mostly from official game keepers (55%), although cases were also submitted by the general public (24%), veterinarians (8%) and the police (4%). Behavioral change was the most common reason for the animals to be culled (60% of all culled cases), followed by fur and skin changes (7%). No history was indicated for the other cases.

### Overview of the main causes of mortality and morbidity

An overview of the main diagnoses is presented in Table 2. Infections were more common (46%) than non-infectious conditions (30%) in all species. The most common infectious conditions were CD in badgers (18% of the submitted badgers) and bacterial infections in martens (16% of the submitted martens), followed by parasitic (4%) and fungal diseases (1%). Infections were followed by trauma (18%), amyloidosis in martens (10% of the submitted martens), and occasional neoplasia and intoxications (both < 1%). There was no conclusive etiological diagnosis for 150 cases (27% of all submissions), including 75 cases of suspected infectious etiology. These were mainly martens and badgers, a few polecats, one weasel and one stoat with bronchopneumonia or interstitial pneumonia (*n* = 29), and/or myocarditis (*n* = 17) and/or hepatitis (*n* = 13) and/or nephritis (*n* = 10) and/or inflammation of the central nervous system (CNS; *n* = 16). All of the animals included in this study that were tested for rabies virus were negative.

**Table 2:** Main etiological diagnoses documented in free-ranging mustelids necropsied at the FIWI, Switzerland, 1958-2015

	Badgers *n* = 249	Martens^a^ *n* = 275	Others^b^ n = 42	Total *n* = 566	% 100
INFECTIOUS	97	66	13	261	46
Viral					
Distemper virus	45	14	1	60	11
Bacterial					
*Streptococcus* sp.^c^	15	15	2	32	6
*Escherichia* sp.	5	9	2	16	3
*Staphylococcus* sp.	–	5	–	5	1
Other bacteria	13^d^	14^d,e^	6^f^	33	6
Fungal					
*Histoplasma capsulatum*	4	–	–	4	1
*Aspergillus* sp.	–	1	–	1	–
Parasitic					
Pulmonary parasites	4	3	2	9	2
Gastro-intestinal parasites	10	–	–	10	2
*Sarcoptes scabiei*	1	5	–	6	1
NON-INFECTIOUS	49	84	7	170	30
Trauma					
Blunt	30	40	3	73	13
Point (bite)	12	8	1	21	4
Nonspecified	–	5	3	8	1
Amyloidosis	1	27	–	28	5
Neoplasia	1	2	–	3	1
Intoxication^g^	1	1	–	2	–
Other non-infectious^h^	4	1	–	5	1
OTHER	49	63	19	167	29
Undetermined^i^	3	43	16	98	17
Non-diagnostic^j^	36	13	3	52	9
Apparently healthy	10^k^	7^l^	–	17	3

^a^Stone and pine marten. ^b^Polecat, otter, weasel. ^c^ *S. canis, S. dysgalagtiae* sp. *S. equisimilis.* ^d^Mixed bacterial infection. ^e^*Pasteurella* sp., *Klebsiella* sp., *Aeromonas* sp., *Francisella tularensis*. ^f^*Mycobacterium* sp., *Pasteurella* sp. ^g^Benzidin and arsenic intoxication. ^h^Myocardial calcification in two badgers, hypertrophic cardiomyopathy in a marten and fatty liver changes in two badgers. ^i^Includes four badgers, 18 martens, a polecat and a stoat suspected to have viral infections and 11 badgers, nine martens and three polecats suspected to have bacterial infections. ^j^Insufficient material quality. ^k^One (1972) and three (1993) badgers of unknown history, three badgers for mange monitoring (2005) and three badgers for targeted testing for *Mycobacterium bovis*. ^l^Martens caught in traps without specified purpose (n = 6) or sent for mange monitoring despite the lack of visible fur changes (n = 1)

The high number of cases submitted in 1993 included mainly culled animals (*n* = 109: 72 martens, 31 badgers, 6 polecats), of which at least 49 cases (45%) were culled due to abnormal behavior (no information available for the others). Main diagnoses in these animals were bacterial infections (*n* = 42, 22% of all cases from 1993), amyloidosis in 26 martens (14% of all cases from 1993) and trauma (*n* = 56, 30% of all cases from 1993).

### Canine distemper

There was a total of 60 CD cases (Table 2), diagnosed by IHC (*n* = 26), PCR (*n* = 17) and/or histology. The death of these animals was always considered directly related to this disease, whether they had been found dead or shot. Shooting was mostly related to behavioral changes such as apathy and daylight activity and, especially in martens, aggression and fearlessness in presence of humans or domestic animals. Animals of both sexes and age categories were affected, without significant differences between these groups.

The majority of CD cases (76%) were diagnosed from 2009 onwards. They included 41 out of 45 CD cases documented in badgers (91%) and 4 out of 11 cases in martens (27%). This corresponded to a significant increase of the percentage of cases affected by CD in both species: Before 2009, CD was diagnosed in only 2% of the submitted badgers and 3% of the martens, while since 2009 CD was found in 60% of the badgers and 27% of the martens (*P* < 0.001 and *P* = 0.016, respectively). From 2009, CD cases in badgers showed a spatial progression from the easternmost part of the country at the border to Liechtenstein and Austria to the west, south-west and north. By contrast, no specific spatiotemporal pattern was observed for CD cases in martens.

**Fig. 4:**
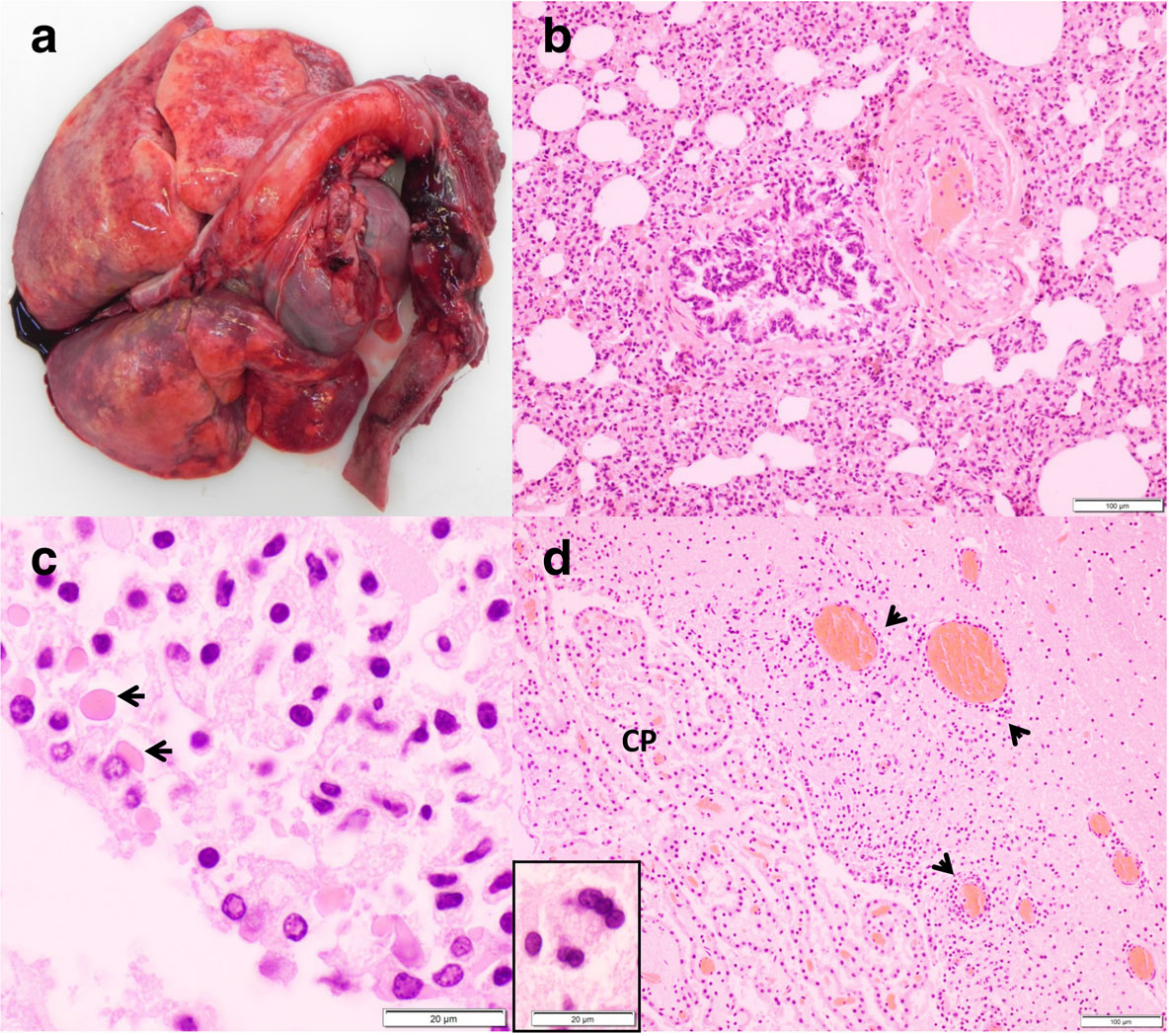
Lesions associated with canine distemper virus infection in badgers. **a** Lungs with “wet-like” appearance, which are not collapsed and display multifocal red to dark red mottling. **b** Lung section, H&E stain: Severe interstitial pneumonia. **c** Brain section, H&E stain: Intracytoplasmic, eosinophilic inclusions (arrows) in the ependymal epithelium. **d** Brain section of the same animal, H&E stain: Multifocal, lymphoplasmacytic perivascular cuffs (arrow heads) and neuronal syncytia (inset), choroid plexus (CP)

Macroscopic lesions associated with CD frequently included patchy areas of dark red mottling in the lungs (70%) and rarely conjunctivitis with mucopurulent ocular discharge (5%) or hyperkeratosis of the foot pads (one badger and one marten). Animals affected by CD were often emaciated (41%) and had no or very little gastrointestinal content. Histologically, the most commonly affected organ systems were the respiratory tract (RT) and CNS, with lesions mostly in both of them (*n* = 31, 51%), less commonly in the RT only (*n* = 26, 43%), and rarely in the CNS only (*n* = 4, 6%). Lesions were characterized by lymphoplasmacytic to histiocytic inflammation in the lungs and CNS (Table 3, Fig. 4a-d). Additionally, hepatic inflammation including hepatitis, cholangio-hepatitis and cholangitis was observed in eight cases (six badgers and two martens from different years). Characteristic intranuclear and/or intracytoplasmic inclusions were reported in 36 cases (60%, including badgers, martens and polecat). Inclusions were found in the bronchiolar, urinary, biliary and gastric epithelial cells in all species, and additionally in ependymal epithelium (*n* = 6, Fig. 4c) and in seminiferous tubules (*n* = 1) in badgers only, and in corneal epithelium and epidermis in one marten each. Syncytia were regularly observed in badgers (in the lung and more occasionally in the brain, Fig. 4d) but only in one stone marten (urinary bladder, Table 3). The histopathological descriptions of the cases for which archival material was not available (cases before 2009 and seven badgers after 2009) were consistent with our own observations in the newly assessed cases.

**Table 3:** Histopathological lesions associated with canine distemper virus infection in free-ranging mustelids, Switzerland, 1958-2015

	CNS						Lung	Syncytia	Inclusions
	Gliosis	Meningitis	Encephalitis	NN	Gray/Both^a^	Ependyma	INT/BINT	L/B	B/L/O
Badger (n = 41)	33 (80%)	21 (51%)	28 (68%)	8 (19%)	16/20	14 (34%)	11/28	16/4	9/24/7^b^
Stone marten (n = 3)	3	2	2	0	0/3	0	3/0	0^c^	4/3/2^d^
Pine marten (n = 2)	2	1	1	0	1/1	1	0/2	0	1/0/0

*CNS* Central nervous system, *NN* Neuronal necrosis, *INT* Interstitial pneumonia, *BINT* Bronchointerstitial pneumonia, *L* Lung, *B* Brain, *O* Other organs

^a^Part of the neuropil affected by the inflammatory process: Gray: gray matter, Both: gray and white matter. ^b^In the epithelial cells of the bile ducts (*n* = 2), urinary bladder (n = 4), stomach (n = 2) and seminiferous tubules (n = 1). ^c^Syncytia formation in one case in the urinary epithelium. ^d^In the corneal epithelium (*n* = 1) and epidermis (n = 1)

Additional infections were reported in 17% of all CD cases and included toxoplasmosis (*n* = 4), streptococcosis (*n* = 2), and one case each of actinomycosis, candidiasis, adiosporomycosis, histoplasmosis and hepatozoonosis. Although there were few cases, toxoplasmosis was diagnosed at a significantly higher proportion among CD cases (4/60, 6.7%) than in the other animals included in this study (2/506, 0.4%; *P* = 0.002).

### Bacterial infections

Bacterial infections were common main diagnoses both in martens and in badgers (15 and 13% of all submitted animals per species, respectively) and suspected in 23 additional cases. Whether as a main diagnosis (*n* = 105) or as an additional finding (*n* = 19), confirmed bacterial infections affected mainly the RT in all species (*n* = 54, 32% of all bacterial infections); infections were associated with lesions consisting of bronchopneumonia or interstitial pneumonia, and, rarely, with pleuritis and pyothorax (n = 5). Other affected organs were the heart (*n* = 37, 22%), kidney (*n* = 20, 19%), liver (*n* = 14, 13%), CNS (*n* = 8, 7%) and skin (*n* = 5, 4%).

*Streptococcus* sp. was the most frequent (n = 19, 35%) isolate in all bacterial RT infections. Ten of 14 badgers (71%) and ten of 20 martens (50%) with *Streptococcus* sp. infection had old skin wounds suspected to have been the portal of entry for the bacteria. Severe, but not specific lesions associated with *Francisella tularensis* in a stone marten (Table 2) have been described in detail elsewhere [58].

Bacterial infections that were not considered to play a role in the main disease process were relatively few (n = 19); they comprised a similar spectrum of organisms as observed for the main diagnoses. They included unspecified *Streptococcus* sp. (two badgers, three martens), mixed flora (four badgers, two martens, one polecat), *Escherichia coli*, *Staphylococcus* sp. and *Actinomyces* (one badger each), *Salmonella* sp. (one stoat) and *Clostridium welchii* (one stone marten). In three cases bacterial species remained undetermined (no growth in culture or bacteria detection at histology). In these 19 animals, the main diagnoses included trauma, CD, histoplasmosis, *Mesocestoides* sp. infection, lymphoma, amyloidosis.

### Fungal diseases

**Fig. 5:**
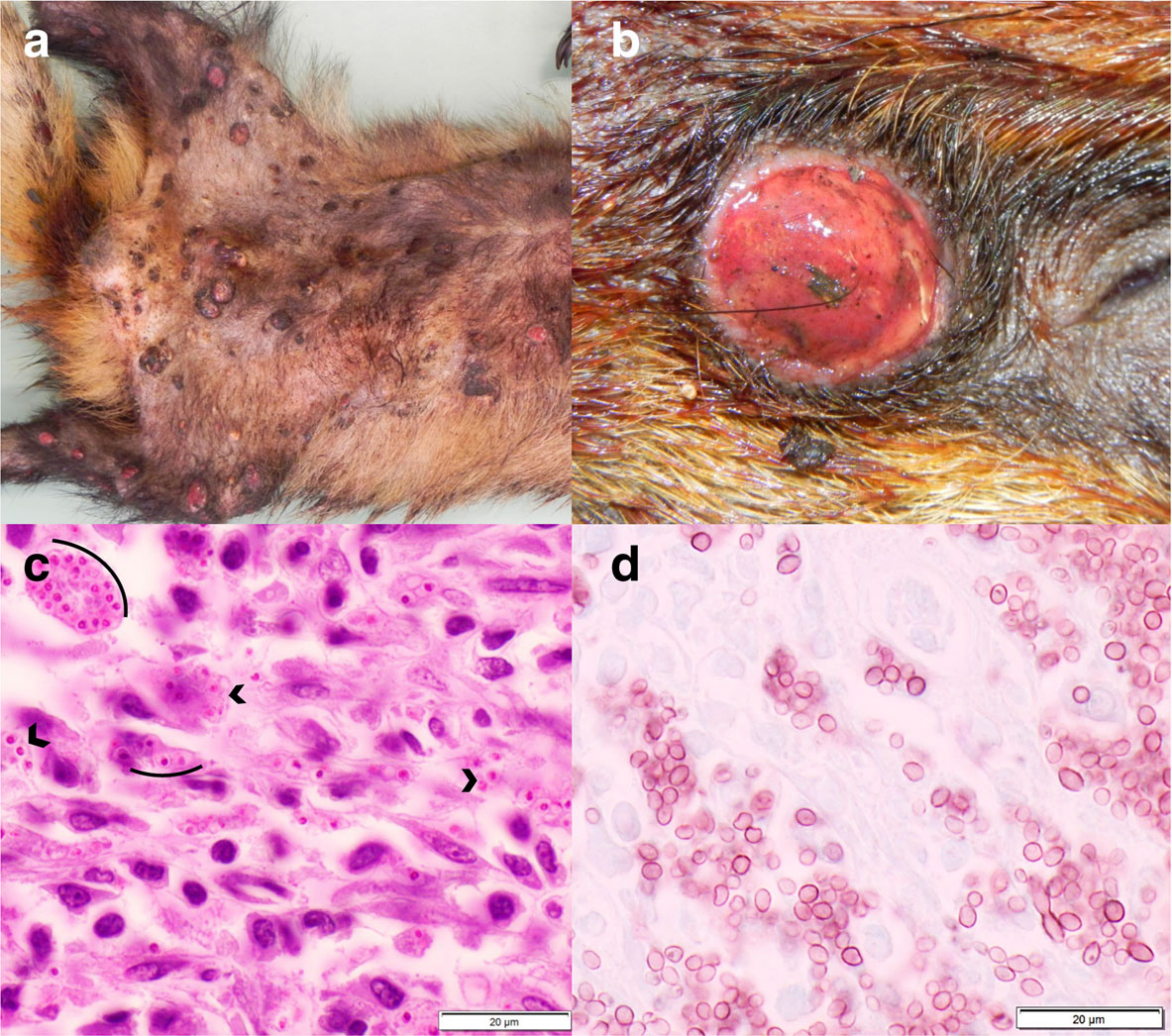
Skin lesions associated with *Histoplasma capsulatum* infection in badgers. **a** Multiple, well-demarcated, ulcerated nodules disseminated on the ventral aspect of the body. **b** Close-up of a nodule. **c** Histological section of a nodule, PAS stain: Numerous round-oval, 1-3 μm diameter yeasts consistent with *Histoplasma capsulatum* (arches). **d** Histological section of a nodule, Grocott stain: Detailed morphology of *Histoplasma capsulatum* with evident narrow-based budding

Mycoses were rarely diagnosed (*n* = 11). Except for four cases of histoplasmosis and one case of aspergillosis (Table 2), they were considered additional findings. Altogether there were six cases of histoplasmosis, a disease which was diagnosed only in badgers. These badgers consisted of five adults (four males, one female) and one juvenile male; they were submitted from various geographical locations and during different years. One of them was also affected by CD. They all had dermatitis, usually suspected to be sarcoptic mange by the submitter. In two badgers the subcutaneous lymph nodes and the lungs were also affected. Lesions were characterized by firm, raised and ulcerated skin nodules associated with hair loss. Similar nodules were also seen in the lungs (Fig. 5a, b). Histologically, the nodules were determined to be granulomas with intralesional fungal elements observed either in the cytoplasm of histiocytes or extracellularly. These organisms were round to oval, 5 μm in diameter, with a 2 μm thick wall, a 2 μm diameter peripherally located round nucleus, and characterized by narrow-based budding (Fig. 5b, d) and morphologically identified as *H. capsulatum*. Macrophages and multinucleated cells, mostly of Langhans type, were the dominant inflammatory cells, followed by lymphocytes and neutrophils.

Other fungal diseases included aspergillosis (in two martens, a badger and a polecat), characterized by granulomatous pneumonia with intralesional fungi, and pharyngeal candidiasis (one badger), characterized by suppurative pharyngitis and tonsillitis with intralesional yeasts.

### Ectoparasites

**Fig. 6:**
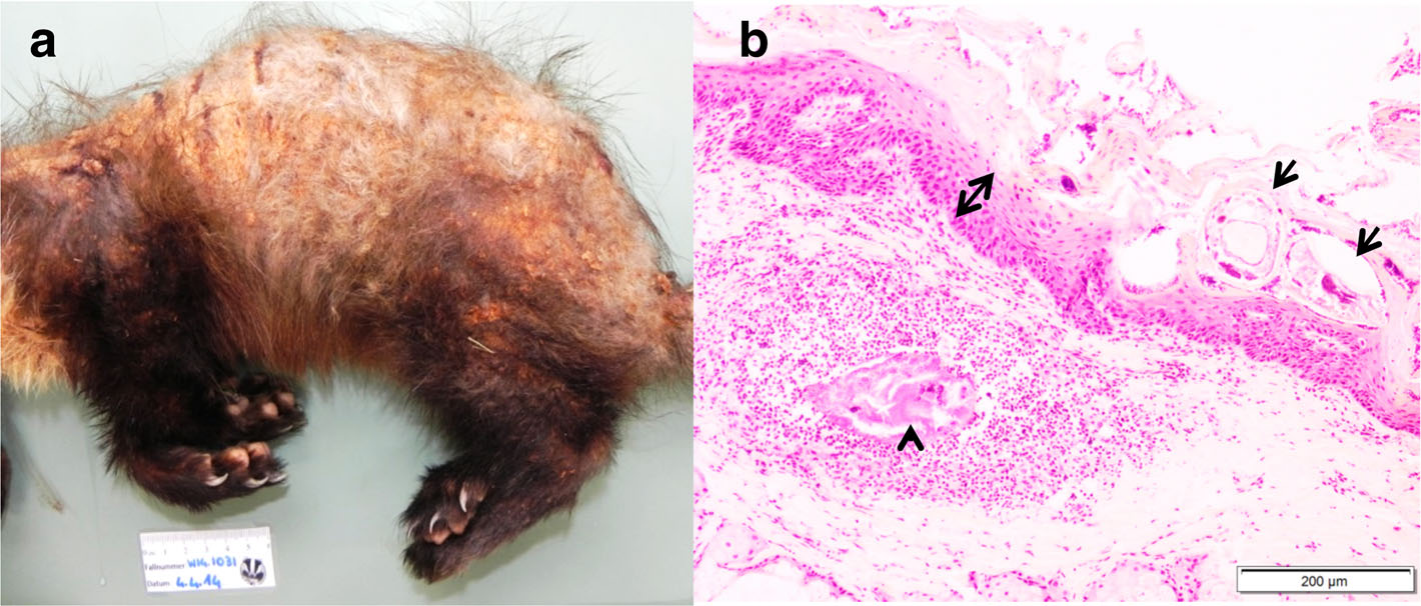
Skin lesions associated with *Sarcoptes scabiei* in martens. **a** Pine marten: Hair loss and skin crust formation affecting mainly the dorsal aspect of the body. **b** Stone marten, histological section (H&E stain): Mites (arrows) embedded in sero-cellular crusts on the hyperplastic epidermis (double headed arrow). Remnants of a mite (arrow head) in the dermis with focal, suppurative dermatitis and dermal edema

Nineteen animals (three martens and 16 badgers) were submitted with a suspicion of sarcoptic mange, which was confirmed in only four of them. Mange was additionally observed in two martens with a different history. Overall, *S. scabiei* was detected in three stone martens (in 2005, 2009 and 2010), two pine martens and a badger (all three from 2014; Table 4). They were all adult animals originating from different geographical locations. In martens, mange was characterized by diffuse crusty skin lesions and hair loss on the dorsal aspect of the body, including the ears, back, tail and occasionally also the flanks, and/or extremities (Fig. 6a). Microscopically there was exuberant hyperkeratosis with cellular debris and bacteria trapped into the thickened keratin, along with moderate epidermal hyperplasia (Fig. 6b). Large numbers of mites were embedded in the crusts or found in the epidermis and rarely the dermis, with moderate to mild infiltration of neutrophils, lymphocytes and plasma cells, and very few mast cells in the superficial dermis. The only badger diagnosed with mange presented with a different picture: Skin lesions were also located on the dorsal aspect of the body but were characterized by hair rarefaction and skin reddening with only minimal crusts on the nose, lumbo-sacral area and lateral thighs. Microscopically there was moderate hyperkeratosis, epidermal hyperplasia and a few mites on the epidermis, with abundant lymphoplasmacytic to histiocytic infiltrations in the dermis; very few eosinophils and mast cells were appreciated.

**Table 4:** Parasites recorded in free-ranging mustelids necropsied at the FIWI, Switzerland, 1958-2015

	Badgers	Martens^a^	Others^b^
ENDOPARASITES			
Pulmonary^c^			
* Eucoleus* sp.	17	7	–
*Crenosoma* sp.	7	6	6
*Filaroides* sp.	14	8	4
* Aelurostrongylus* sp.	2	–	–
*Angiostrogylus* sp*.*	1	–	–
Gastrointestinal^d^			
Nematodes			
*Capillaria* sp.	15	35	2
*Uncinaria* sp.	9	5	1
Ascarids	2	–	1
Trichostrongylids	3	5	4
Cestodes	39	18	2
*Atriotaenia incisa*	15	–	–
*Taenia* sp.	6	13	1
Trematodes			
*Euryhelmis squamula*	2	–	–
Protozoa			
*Isospora* sp.^e^	24	3	–
*Eimeria* sp.	1	–	1
*Cryptosporidium* sp.	1	–	–
Muscular			
*Trichinella* sp*.*	–	2	–
*Sarcocystis* sp.	1	1	–
*Hepatozoon* sp.	1	3	–
*Alaria* sp.	–	1	–
Multisystemic			
*Toxoplasma gondii*^f^	1	3	1
*Mesocestoides* sp.	1	1	–
ECTOPARASITES			
Ticks *(Ixodes* sp*.)*	15	8	4
Fleas *(Paraceras* sp*.)*	5	7	1
Lice *(Trichodectes* sp.*)*	45	–	1
Mites			
*Sarcoptes scabiei*^g^	1	5	–
*Demodex* sp.	1	–	–

Parasites detected either at macroscopic pathological examination or by coprology. Since the exact number of analyses is not known for the different parasites, no percentage was indicated. ^a^Stone and pine marten. ^b^Polecat, otter, weasel. ^c^Clinically relevant in four badgers, three martens and two polecats. ^d^Clinically relevant in 10 badgers. ^e^Identified as *I. melis* (six badgers), *I. mustelae* and *I. lacazei* (three and one marten, respectively). ^f^ Clinically relevant in a marten and a polecat. ^g^All clinically relevant

**Fig. 7:**
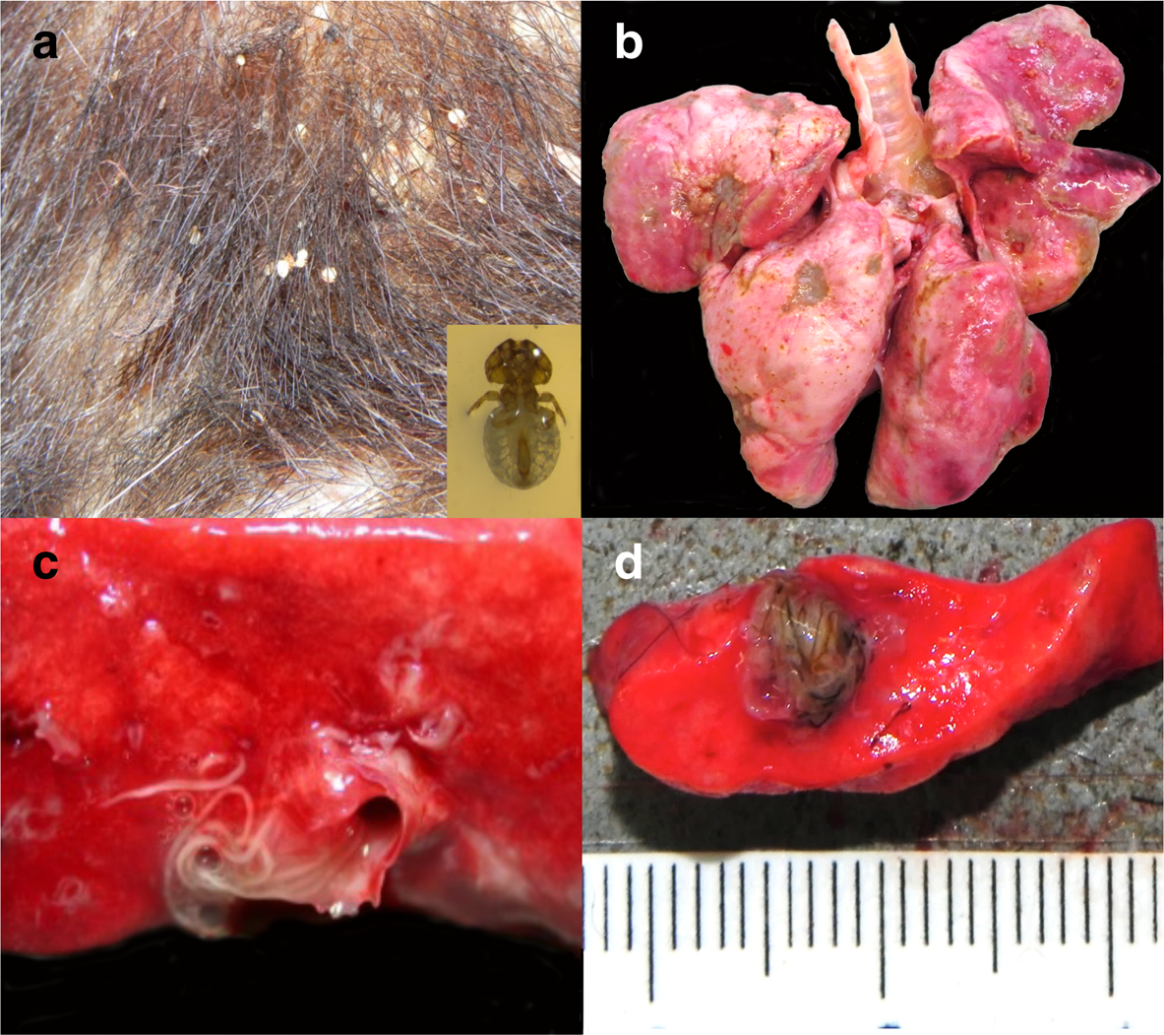
Common ecto- and endoparasitism in free-ranging mustelids from Switzerland. **a** Close-up of the skin of a badger with severe *Trichodectes melis* infestation with hair loss and crust formation. A close up of *T. melis* is shown in the inset. **b** Lungs of a badger with multifocal, light tan to gray indented areas associated with *Crenosoma* sp. **c** Cut surface of the lungs of a badger with numerous intrabronchial lungworms, *Crenosoma vulpis*. **d** Cut surface of a polecat’s lung with a cluster of lungworms, *Filaroides* sp.

Of a total of 16 badgers with alopecic lesions, 11 (69%) were infested by biting lice and/or ticks (Fig. 7a, Table 4). In these cases, alopecia was especially prominent on the ventral aspect of the body. By contrast, lice were never reported in martens. Focal alopecia and/or crust formation were typically attributed to mange by the submitters, but these lesions sometimes resulted from bite wounds, which were another common cause of skin lesions both in badgers (*n* = 20) and martens (*n* = 26). In one badger with bite wounds and severe lice infestation, histological examination revealed the presence of mites consistent with *Demodex* sp. in association with pustular dermatitis.

### Endoparasites

The exact number of mustelids for which a parasitological examination was conducted was unclear and semi-quantitative data were mostly lacking, but endoparasites were mentioned in 239 (42%) of the submitted cases. The reported parasites consisted mainly of helminths detected in the digestive tract (124 cases or 81% of all mustelids with gastrointestinal parasites) and/or in the lungs (96 cases or 97% of all mustelids with pulmonary parasites, Fig. 7b-d). Intestinal protozoans (mainly *Isospora *sp.) were repeatedly found in badgers but were uncommon in martens (Table 4). The intestinal cestode *Oochoristica incisa* (Syn: *Atriotaenia incisa*) was mentioned 15 times in badgers until 1992 but it was not found in other mustelid species and was not mentioned in later years anymore. Rarely observed lung or gastro-intestinal parasites included *Aelurostrongylus* sp., *Euryhelmis squamula* and *Cryptosporidium* sp., which were reported only in badgers (Table 4).

Gastro-intestinal parasites were reported to be associated with a local inflammatory reaction, mostly lymphoplasmacytic to eosinophilic, in only 10% of the infected animals. Lungworms were reported to be associated with a local inflammation in 75% of the cases, which consisted mostly of lymphoplasmacytic to histiocytic inflammatory reaction and increased alveolar macrophages. Lesions associated with *Filaroides* sp., one of the more commonly mentioned lungworms in mustelids, were characterized by nodules of 5-10 mm in diameter containing numerous tightly whorled, gray to green nematodes (Fig. 7d).

Other parasites were located intramuscularly (cardiac and skeletal musculature) and included *Hepatozoon* sp. (in three martens, associated with mild granulomatous myocarditis; and a badger with CD, without *Hepatozoon* sp.-associated inflammation), *Trichinella* sp., *Alaria* sp., and *Sarcocystis* sp. in martens (one case each) without inflammation. *Toxoplasma gondii* cysts were found histopathologically in brain or lung of four martens and a badger and in multiple organs of a polecat, of which three martens and the badger had CD. Although very rarely mentioned (*n* = 2), larval *Mesocestoides* sp. was abundant in one marten with numerous parasite cysts in the abdomen and thorax with mild lymphohistiocytic serositis. In one polecat, unilateral bone penetration in the infraorbital region suggested *Troglotrema* sp. infestation but the parasite itself was not detected.

### Amyloidosis

Amyloidosis was a frequent non-infectious condition observed in this study (57 cases) and it affected martens (55/275, 20%) significantly more often than badgers (2/249, 1%; *P* < 0.001). Amyloidosis has been regularly reported since 1965, either as a main diagnosis (Table 2) or as an additional finding. It was detected more frequently in adults (44/172, 26%) than juveniles (4/55, 7%; *P* = 0.004). There was no difference between sexes (31/106 or 29% adult males vs. 13/66 or 20% adult females; *P* = 0.209). In martens, amyloidosis was frequently the main pathological process, without any predisposing chronic inflammation (*n* = 32, 58% of all marten cases with amyloidosis). Bacterial infections and endo−/ectoparasitism were significantly less frequent in martens with amyloidosis than martens without it (42% vs. 89%; *P* < 0.001). One of the two badgers with amyloidosis had CD but the other case presented with only an intestinal *Isospora melis* infection without associated lesions.

**Fig. 8:**
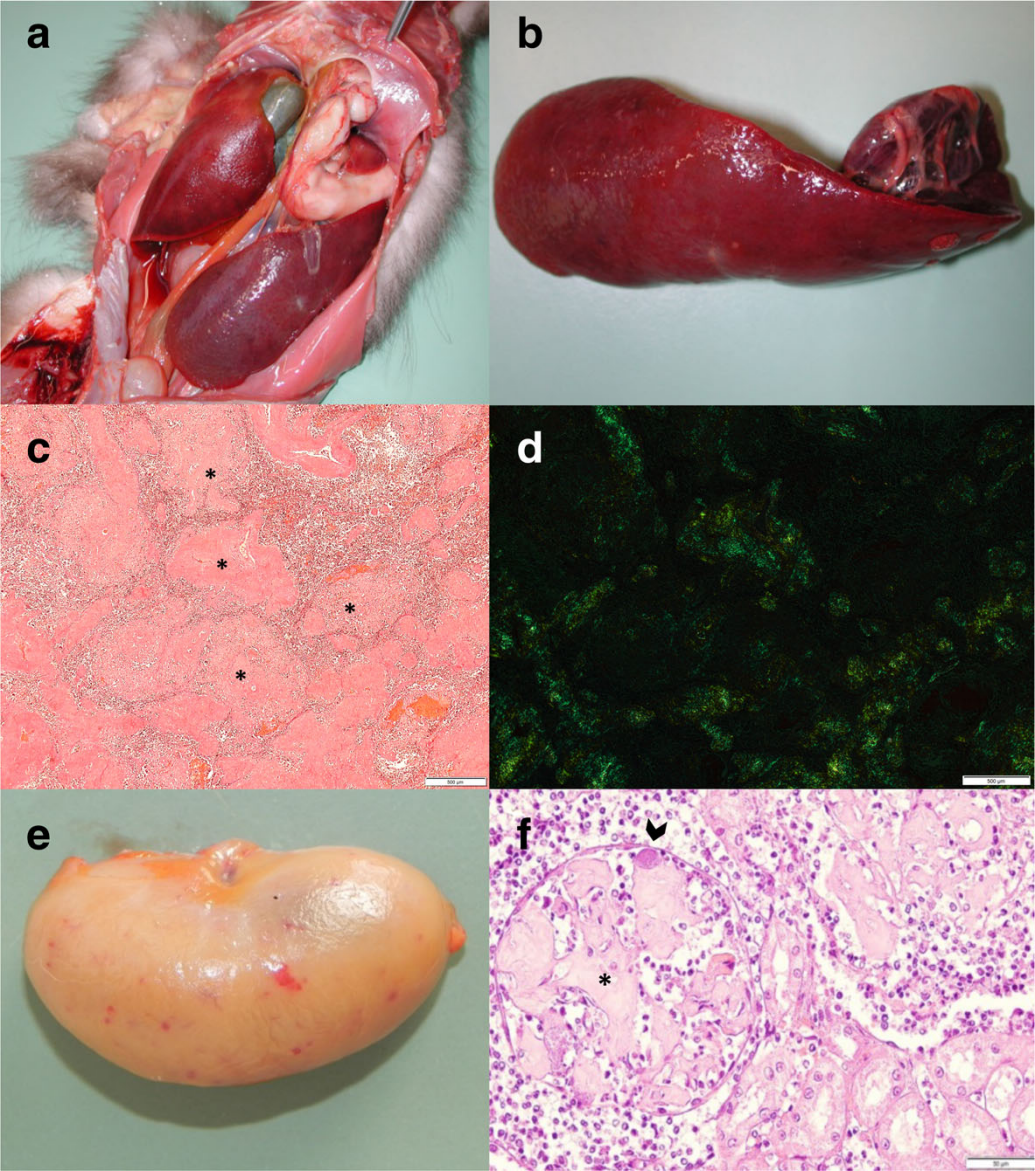
Lesions associated with amyloidosis in martens. **a** Pine marten, abdominal organs *in situ*: Severely enlarged spleen. **b** Same pine marten as in a, extracted spleen: Severe splenomegaly, increased consistency and rubber-like texture. **c**) Stone marten, Congo red stain, spleen: Severe deposition of amyloid (asterisk) around the blood vessels. **d** Same stone marten as c, Congo red stain, spleen: Apple-green birefringence under polarized light. **e** Pine marten, kidney: Diffuse tan discoloration with petechial hemorrhages. **f** Stone marten, histological section of a kidney (H&E stain): The glomeruli are partially obstructed by abundant deposit of eosinophilic, fibrillary material consistent with amyloid (asterisk) and a bacterial embolus within the lumen of the glomerular capillary (arrow head)

Amyloidosis was multisystemic in most cases (66%), although the spleen was most commonly affected (94%), followed by lesions in the kidneys (45%), liver (42%), heart (24%) and mesenterial lymph nodes (one marten). Macroscopically, lesions were splenomegaly together with a rubber-like consistency (Fig. 8a and b), and a slight enlargement of the kidneys rarely in combination with pallor and petechiae (Fig. 8c). Histologically, deposits of a homogenous, eosinophilic, glassy material (amyloid) were found perivascularly in the spleen (Fig. 8d, e); in the basement membrane of the glomerular tufts, Bowman’s capsule and less commonly in the renal tubules (Fig. 8f); within the spaces of Disse and portal areas of the liver; within the heart valves; and perivascularly in several other organs. The brain was investigated in four martens but no lesions were detected.

The retrospective evaluation of tissue sections of eight martens revealed non-AA amyloidosis in five cases and AA-amyloidosis in three.

### Other lesions of non-infectious origin

Traumas were most common in adult animals (74% of all traumas) both in badgers (*n* = 44; 29% of all adult badgers) and martens (*n* = 51; 20% of all adult martens). Blunt traumas variably affected the head, thorax, or hind limbs. Bite wounds were mostly found in the frontal part of the body including neck, ears and shoulders (66%), and at the base of the tail (18%).

Neoplastic conditions were of major clinical relevance in only three cases, including lymphoma in a badger and stone marten and non-specified abdominal masses in a pine marten (Table 2). Other neoplasms were classified as side pathological processes and included a non-specified benign ovarian tumor, a dermal hemangioma and a pulmonary mesenchymal tumor in badgers (one case each) and non-specified masses in the liver and spleen in martens (one case each).

Tooth wear, and missing or broken teeth, were repeatedly reported in adult badgers (*n* = 19), once in association with severe osteomyelitis of the mandible. Pneumoconiosis was also observed multiple times and mainly in badgers (25 badgers and three martens) throughout the years 1964 to 2015.

## Discussion

Our study provides a first overview of diseases and causes of death diagnosed in free-ranging mustelids in a continental European country. The number of submitted cases represents a minor portion of the dead mustelids recorded by the Swiss hunting authorities (< 0.3% of the carcasses have been submitted for analysis per year in the past 20 years) (Fig. 2). However, the species representation in our study generally reflected their occurrence and abundance in the wild, with the exception of stoats. Stoats were submitted less frequently than polecats although stoats are more abundant than polecats in Switzerland [59]. The proportionally very low number of stoats might be related to their small body size, as carcasses of small wildlife species are less likely to be detected [60, 61]. However, in a similar study conducted in Great Britain [31], stoats were proportionally more numerous than polecats in the submitted material. This suggests that other factors must influence the detection and submission of stoat carcasses, such as their likelihood to be found in the environment or the public interest for these animals.

Cases originated from all parts of the country, but predominantly from the canton of Bern and neighboring regions (Fig. 3) located in relatively close proximity to the FIWI. Nevertheless, considering that mustelids can easily be shipped by regular mail thanks to their small body size, the geographical proximity alone does not explain this bias. The uneven distribution of cases is likely due to the fact that not all Swiss cantons have a long tradition of wildlife health surveillance and that until the 1990s most wild animals found in western Switzerland were submitted to another laboratory [41, 43]. The higher numbers of cases submitted from the canton of Basel-Stadt and Basel-Landschaft (Fig. 3) were mainly martens. These two cantons have the highest (Basel-Stadt: 5072 inhabitants/km^2^) and fourth highest human density (Basel-Landschaft: 502 inhabitants/km^2^) of all Swiss cantons. Stone martens are known as “culture chasers” due to their ability to take advantage of human infrastructure, such as deserted buildings, roof floors and bridges that they use as shelter [62–64]. Therefore, in such an environment, dead martens and/or animals observed with disease signs such as abnormal behavior or skin lesions are more likely to be found, and the incentive for submission to a laboratory may be higher due to the fear of pathogen transmission to domestic pets or humans.

Behavioral change was indeed the most common reason for case submission. Rabies was frequently diagnosed in mustelids as well as in other carnivores by the Swiss Rabies Center from 1967 until eradication in 1997 (Fig. 2) [65, 66], and many of the mustelids submitted for necropsy at the FIWI [this study] were diagnosed with either CD or amyloidosis. Rabies and CD are well known to be associated with behavioral changes, and amyloidosis has also been reported to be associated with aggression and fearlessness in martens [52].

The case load per year was relatively stable (Fig. 2), except for the marked peak in 1993, which was most likely related to the increased awareness of the public due to the rabies control efforts in Switzerland in the early 1990s. Despite a history of abnormal behavior, neither rabies nor CD was diagnosed in any of these cases. Causes of mortality included mainly bacterial infections, blunt trauma and amyloidosis, in proportions comparable to those obtained for the whole study period. Regarding the unclear cases with suspected CD lesions that were retrospectively tested by PCR, degradation of the RNA in the old paraffin blocks may have impaired the detection of CDV.

Given that the majority of submitted samples were adults, this study delivers mostly data for this age group. The low number of juveniles may be due to a lower likelihood of being detected due to their smaller body size and/or to their denning behavior from birth in early spring to dispersion in mid-autumn [67]. Similarly, higher numbers of cases in spring and summer might be associated with a higher mortality rate related to a higher biological activity of mustelids during this period of the year [68]. Such an increase in case observations in the spring was indeed previously observed in badgers with bovine tuberculosis [4].

In the majority of our cases, the main diagnosis was of infectious origin. This contradicts another retrospective study on mustelids by Simpson et al. [31] and the national hunting statistics [69]. This difference is likely due to the fact that animals found dead in the field with a clear picture of trauma such as a car collision are generally not submitted for post mortem investigation in Switzerland and are therefore underrepresented in our material. By contrast, Simpson et al. [31] specifically collected animals killed in traffic accidents or trapped in the framework of a pest control program to conduct their study. Nevertheless, trauma remained the most common non-infectious cause of death in our study, and bite wounds were suspected to have played a role in infectious disease cases by causing a port of entry for opportunistic bacteria.

The data available for this study were collected in the framework of routine examinations, i.e., investigations were not done systematically according to a predetermined protocol but were question-oriented. However, macroscopic examinations were performed according to institute standards, and histology and ancillary tests were frequently performed. Only selected organs were submitted for less than 3% of the animals. Overall, it seems unlikely that major information on the cause of mortality and morbidity of the submitted animals would have been lost in relation to the study design.

### Canine distemper

Distemper is a well-known disease in mustelids [8–11, 70, 71], and since the eradication of rabies it has become the main viral disease affecting mustelids in Switzerland. The emergence and distribution of badger cases from 2009 onwards indicates a relationship with the national epidemic in the red fox, as also shown by molecular analyses in Switzerland and other countries [10, 72–74]. By contrast, the situation in martens is less clear. A link between the fox epidemic and marten cases after 2009 seems likely, considering that the virus was found in other hosts during this period, including a free-ranging lynx (*Lynx lynx*), a domestic dog (*Canis familiaris*) [10], a domestic cat (*Felis catus*) [75] and captive Asian marmots (*Marmota caudata*) [76]. However, CD was present in stone martens in Switzerland before this epidemic [63; this study] and there were similarities between the older and recent strains [10], suggesting that martens might play a role as reservoir of CD virus. Nevertheless, the more common occurrence of CD cases in badgers compared to martens might be related to different risks of intraspecies transmission due to different social systems, as badgers live in social groups whereas martens are solitary animals [68].

The pathological picture of CD in our study animals corresponds to former reports of CD in mustelids [10, 70]. Differences among histological lesions in red fox, badgers and martens were previously pointed out [10], however, this observation was based on the examination of only eight badgers and three martens. Here we confirmed the existence of species-specific differences: Periventricular and ependymal CNS lesions were found only in badgers and syncytia formation in brain and lung were not found in martens, which might suggest a different host-related pathogenesis of the disease [10, 77]. Concurrent diseases like toxoplasmosis and secondary bacterial infections might have been favored by CDV-induced immunosuppression [78, 79]. However, since this situation concerned only a minority of cases in our study and these diseases were also found in CDV-negative animals, concurrent infections may have been purely incidental.

### Bacterial infections

Bacterial infections were mostly associated with pulmonary or cutaneous disorders and typically involved *Streptococcus* and/or *Staphylococcus* species. These bacteria were reported as the most common pyogenic bacteria in mustelids [52] and are known to be associated with dermatitis, respiratory infection, inflammation in several organs and septicemia in various animal species [52, 70, 80, 81]. In our study, many animals with bacterial infections had skin wounds. Since *Streptococcus* sp. and *Staphylococcus* sp. belong to the mucosal microflora [82], the skin wounds were suspected as a portal of entry for the bacteria, as previously suggested for martens [52], otters [83] and foxes. Other less common bacterial pathogens like *Pasteurella* sp. [31, 84], *Klebsiella* sp., *Clostridium* sp. and *Francisella tularensis* [84] were detected in our study in agreement with previous reports.

The typical inflammatory pattern associated with aerogenic bacterial infections in the lungs is bronchopneumonia [85]. However, in our study several animals with bacterial infections had an interstitial pneumonia. This might be due to a primary or concomitant viral infection, or to a vascular spread of the bacteria from another portal of entry (such as a skin wound) to the pulmonary interstitium, rather than a primary lung infection through inhalation. Finally, frequent occurrence of bacterial bronchopneumonia with concomitant lungworm infection suggests that tissue damage by lungworms may predispose to bacterial infection [86].

Infections with *Mycobacterium bovis* are frequent in Eurasian badgers in several countries. In the United Kingdom, badgers have played a role as a reservoir for *M. bovis* for decades [4, 6, 87] and there is some concern that this species could maintain *M. bovis* in other countries such as France or Spain [88, 89]. Bovine tuberculosis (bTB) has re-emerged in Europe, including countries surrounding Switzerland [90, 91], but none of the causative agents (*M. bovis* and *M. caprae*) has been identified in Swiss wildlife since the country was declared officially free of bTB in 1960 [90]. Switzerland has been repeatedly mentioned as the first country with bTB diagnosis in a badger but these statements relied on an incorrect citation of an old article reporting a bTB case in an Alpine chamois (*Rupicapra rupicapra rupicapra*) in 1951 [92]. Although targeted investigations have not been performed in badgers so far, to our knowledge, bTB has never been diagnosed in badgers in Switzerland.

### Skin lesions

The second most frequent reason for submission after abnormal behavior was skin changes, mostly assumed to be mange by the submitter. Since 2002, the FIWI has run a nationwide monitoring program for sarcoptic mange [93] and actively asked field partners to submit all wild mammals showing mange-like lesions for analysis. However, mange was diagnosed in only a few mustelids. Skin lesions were mostly related to other causes like infected bite wounds, histoplasmosis, and severe lice or flea infestation.

Sarcoptic mange is a contagious skin disease affecting numerous mammal species and typically associated with high mortality in wildlife [94]. In Switzerland, it has been present for several decades in wild carnivores, progressively expanding all over the country. It kills mainly foxes [95] and occasionally other species such as the Eurasian lynx [96] and martens (this study), all showing severe crusty lesions consistent with the fatal form of the disease [95]. Despite the many badgers with skin lesions that were examined for mites, *S. scabiei* was detected in only one badger in our material. Previous studies [32, 97, 98] have also pointed at the rarity of sarcoptic mange in badgers. The pathological picture in the single badger found infested with mites was largely consistent with the alopecic form of mange in the healing phase, except for the presence of a few mites in the lesions [95]. Overall, the rare occurrence of mange cases in badgers and the mild signs recorded in this affected individual suggest that badgers are less susceptible to *S. scabiei* than other carnivores. This is also supported by the comparison of the number of mange and CD cases among foxes, lynx, martens and badgers during the past ten years, when these two contagious diseases became widespread in carnivores in Switzerland: Cases were numerous in foxes and low in martens and lynx, but in all three species there was a similar proportion of mange and CD cases [FIWI unpublished data, this study], hinting at a comparable spread and clinical impact of the two causative agents in these hosts. By contrast, in badgers the proportion of mange was very low compared to CD.

In contrast to putative sarcoptic mange susceptibility, badgers were commonly infested with biting lice, while these ectoparasites were not noticed in martens. Lice were repeatedly associated with alopecia in badgers, and it is conceivable that pruritic licking and scratching causes these skin changes. Indeed, alopecia associated with lice was typically distributed on the ventral aspect, where the parasite is commonly found, as opposed to the dorsal lesions characterizing sarcoptic mange.

Histoplasmosis was diagnosed only in badgers, with a pathological picture corresponding to previous reports in this species [25–28, 50]. *Histoplasma capsulatum* occurs in soil contaminated with bird and bat droppings and is known to affect mainly dogs but also many other mammals, including humans [50, 99]. Susceptibility of the badger to this zoonotic disease was proposed to be related to their omnivorous nature [100] or to selective immunodeficiency due to evolutionary and immune system development [26].

Bite wounds were repeatedly observed both in martens and badgers and identified as the primary cause of skin lesions such as post-scarring alopecia. Bite wounds are common in mustelids [83, 101–103] and may be related to intraspecific fights, especially in martens. In mustelids there is a correlation between the degree of intra-sexual territoriality (associated with aggressive behavior) and the degree of body elongation, sexual dimorphism and degree of carnivorous behavior [104], three characteristics which are more pronounced in martens than in badgers.

### Endoparasites

A wide range of endoparasites was documented in this study. Part of these were previously reported in badgers, martens, polecats and/or minks, including gastrointestinal parasites such as *Isospora *sp., *Eimeria* sp., *Uncinaria* sp., *Taenia* sp., *Capillaria* sp. and *Euryhelmis squamula* [19–22, 105–107], muscle parasites like *Trichinella* sp. [108–110], *Alaria* sp. [111] *Hepatozoon* sp. [24] and *Sarcocystis* sp. [112], and systemic parasites like *T. gondii* [113]. Larval infection with *Mesocestoides* sp. is known to be of zoonotic importance as it has been reported in humans [114], dogs and other canids [115], and in badgers and martens [52, 111]. *Oochoristica incisa* (*Syn. Atriotaenia incisa*) was also previously described as a common enteral cestode in badgers [20, 116] but it was not reported in the necropsy reports of the FIWI after 1992. This is likely a bias due to a change in investigation techniques, because after 1992 gastrointestinal contents was no longer routinely washed out and sieved to retrieve adult parasites. Similarly, widespread gastro-intestinal helminths of roe deer were no longer documented at the FIWI in the past two decades [43]. Furthermore, eggs of trichostrongylid-type (not further identified) that were found in this study could indicate the sporadic presence of non-mustelid specific parasite stages (Table 4). Possible shedding of ingested parasite stages should be taken into account when performing coprological analyses for endoparasites of carnivores [117]. Lungworms have been repeatedly reported in mustelids [19, 21, 23, 31], with mostly the same species as in our study, including *Capillaria aerophila* (*Syn. Eucoleus aerophilus*), *Crenosoma*, *Filaroides* and *Aelurostrongylus* species. By contrast, *Skrjabingylus* sp. was reported in various European countries [21, 31, 118–120] but was not detected in the mustelids present in this study. *Skrjabingylus* sp. is a parasite of the nasal cavity, a body localization that is rarely examined, and it may have been missed during routine examination. *Angiostrongylus* sp. was only detected in one badger in our study, but had been previously reported not only in badgers [10, 20] but also in stoats and weasels [31, 121].

Although very commonly observed in our study, endoparasites seem to be of minor clinical relevance, in agreement with previous studies which documented numerous parasite species in apparently healthy mustelids [19–21]. Nevertheless, they were identified as being the main etiological diagnosis in 19 animals in this study (Table 2). Furthermore, while gastrointestinal parasites were only occasionally associated with degenerative changes (mucosal hyperplasia) and/or inflammation, pulmonary parasites were commonly documented with associated inflammation. This reaction was mostly mild but as stated above, it may have predisposed the affected hosts to secondary bacterial infection.

### Amyloidosis

Amyloidosis is a well-known and frequent disease entity in martens. Because the etiology is mostly unknown, it is often referred to as “idiopathic amyloidosis” and considered to be a metabolic disorder [29, 30, 52, 122]. The term amyloidosis refers to the accumulation of a misfolded protein called amyloid. Two types of amyloidosis are distinguished in animals: AA- (or secondary amyloidosis) and non-AA amyloidosis. AA amyloidosis is the most common type and is related to chronic inflammation. Non-AA amyloidosis includes different forms, determined by the biochemical nature of the accumulating proteins. Among them, AL amyloidosis (or primary amyloidosis) is the least common form; the amyloid proteins are derived from immunoglobulin light chains [123, 124]. AA and non-AA amyloidosis are characterized by the same pathological picture; Congo red staining with potassium permanganate pretreatment is required to distinguish them on histopathology [56]. In our material there was no association between amyloidosis and chronic infections, and amyloidosis appeared to be a primary pathological process. However, this and previous studies have demonstrated both AA and AL amyloidosis in martens [29, 30, 122, 125], suggesting that in martens amyloidosis cannot be attributed to a single cause. Genetics has been proposed to play a role in black footed ferrets [30] and stone martens [126], however, evidence of the transmissibility of the amyloid protein has been recently shown in laboratory animals [127, 128]. It has also been hypothesized that amyloidosis may be secondary to Aleutian disease (AD) [129] in a case presenting with increased lymphoid reaction, hypergammaglobulinemia, glomerulonephritis, periportal hepatitis and vasculitis, which are features potentially caused by AD [73, 130–134]. Exposure to AD parvovirus has been shown by serological methods in captive and free-ranging mustelids in several European countries [13–15, 17, 18] but so far similar investigations have not been performed in free-ranging mustelids in Switzerland. Since AD is known to be associated with meningoencephalitis, this disease should also be considered to be one of the differentials in the mustelids of this study, in which CNS inflammation was recorded but no etiological diagnosis was achieved.

Amyloidosis was commonly associated with behavioral changes in martens. Since the brain of the affected animals has only rarely been investigated histologically, it is not possible to assess the existence of amyloid deposits in this organ. However, this was not found in the samples we investigated, and non-specific behavioral changes may have been induced by the effects of systemic disease in the absence of CNS lesions.

### Other lesions

We repeatedly observed oral lesions involving the teeth in badgers. Dental problems are well known in this species, especially in old individuals, and may be related to severe dental wear resulting from species-specific foraging behavior, such as the habit of digging into the ground to search for one of the badgers’ favorite prey, earthworms [133, 134]. This might also explain the pneumoconiosis, which was more common in badgers than martens in our study.

## Conclusion

Infectious diseases were common in mustelids from Switzerland during the whole study period, but their etiology varied over time. Strong similarities were noticed between disease occurrence in mustelids and the disease patterns found in red foxes: Rabies was frequently reported in mustelids prior to eradication in 1997, CD was often diagnosed beginning with the nationwide epidemic in 2009, and sarcoptic mange was sporadically observed for the past 15 years. This suggests pathogen transmission from foxes to other carnivores. Other noteworthy diseases diagnosed over the whole study period included bacterial infections mainly due to *Streptococcus* sp. and helminthiasis, as well as a few cases of hepatozoonosis, toxoplasmosis, aspergillosis and histoplasmosis. Overall, lung pathologies of infectious origin were very common, in agreement with former observations [31]. We also documented numerous differences between badgers and martens regarding disease etiology, frequency of disease occurrence and the type of lesions for the same disease. Importantly, we found no indication that mustelids currently play a role as reservoir for zoonotic pathogens in Switzerland. These data can serve as a baseline for future general health surveillance in Switzerland and for comparison with other European countries.

## Abbreviations

CD: Canine distemper
CDV: Canine distemper virus
CNS: Central nervous system
FIWI: Centre for Fish and Wildlife Health
IFA: Immunofluorescence assay
IHC: Immunohistochemistry
PCR: Polymerase chain reaction
RNA: Ribonucleic acid
RT: Respiratory tract
RT-PCR: Reverse transcription polymerase chain reaction
